# Functional analysis of *CgWRKY57* from* Cymbidium goeringii* in ABA response

**DOI:** 10.7717/peerj.10982

**Published:** 2021-02-23

**Authors:** Huanhuan Liu, Lianping Wang, Xijun Jing, Yue Chen, Fengrong Hu

**Affiliations:** 1 College of Landscape Architecture, Nanjing Forestry University, Nanjing, Jiangsu, China; 2 Institute of Horticulture, Zhejiang Academy of Agricultural Sciences, Hangzhou, Zhejiang, China

**Keywords:** *Cymbidium goeringii*, WRKY genes, ABA stress, Transgenic

## Abstract

**Background:**

The orchid is one of the top ten Chinese flowers and has high ornamental value and elegant color. However, orchids are vulnerable to abiotic stresses during their growth and development, and the molecular mechanism of the abiotic stress response in orchids is unclear. WRKY proteins belong to a transcription factor family that plays important roles in biotic stress, abiotic stress, growth and development in plants, but little is known about the WRKY family in *Cymbidium goeringii*.

**Methods:**

The specific fragment of the *CgWRKY57* gene of *C. goeringii* was analyzed by bioinformatics. The expression of the *CgWRKY57* gene of *C. goeringii* under 4 °C, 42 °C water and ABA stress as well as different tissues was detected by real-time fluorescence quantitative PCR. *CgWRKY57* gene was overexpressed in wild type *Arabidopsis thaliana* by inflorescence infection method, and the function of transgenic lines under ABA stress was analyzed.

**Results:**

*CgWRKY57* was cloned from *C. goeringii* and found to encode 303 amino acids. The CgWRKY57 protein is an acidic, nonsecreted hydrophilic protein without a signal peptide or transmembrane domain. The CgWRKY57 protein is located to the nucleus and may function intracellularly according to its predicted subcellular localization. A domain analysis and homology comparison showed that the CgWRKY57 protein has a “WRKYGQK” domain and belongs to Group III of the WRKY family, and a phylogenetic analysis demonstrated that CgWRKY57 is closely related to OsWRKY47. *CgWRKY57* was expressed in the roots, stems, leaves and floral organs of *C. goeringii*, and its expression level was highest in the roots according to real-time qPCR analysis. There were significant differences in *CgWRKY57* expression under 4 °C, 42 °C ABA and water stress treatments, and its expression changed greatly under ABA stress. The expression of *CgWRKY57* in transgenic plants was significantly higher than that in wild type plants under ABA stress, and the root length and germination rate were reduced in transgenic plants compared to wild type plants.

**Conclusions:**

These results indicate that *CgWRKY57* overexpression is responsive to ABA stress, and they provide a foundation for future analyses of the biological functions of the WRKY family in *C. goeringii*.

## Introduction

WRKY transcription factors (TFs) are vital regulatory proteins and are involved in regulating responses to abiotic stress in plants (Rushton et al., 2010). The first *WRKY* gene was identified from *Ipomoea batatas* in 1994 (Ishiguro & Nakamura, 1994). Two WRKY proteins, ABF1 and ABF2, were identified from *Avena fatua* and their DNA-binding regions had a Zinc-finger motif (Rushton et al., 1995). WRKY proteins have a highly conserved WRKY domain, but these proteins differ in size. The WRKY domain is composed of 60 amino acid residues and has highly conserved “WRKYGQK” sequences in the N-terminal region and zinc finger domains (C-X4-5-C-X22-23-H-X1-H or C-X7-C-X23-H-X1-C) in the C-terminal region (Eulgem et al., 2000). WRKY proteins bind to the *cis*-acting element W box in the promoters of target genes to regulate stress-related gene expression, allowing plants to achieve stress resistance (Ciolkowski et al., 2008).

Currently, the classification of the WRKY family is based on the genomic characteristics of the *Arabidopsis* WRKY family (Eulgem et al., 2000). The WRKY family is divided into three groups (I, II and III) according to the number of domains and the types of zinc finger domains. Group I has two WRKY domains and two types of zinc finger domains, while Groups II and III have one WRKY domain per protein. The zinc finger domains of Group II are identical to those of Group I (C-X4-5-C-X22-23-H-X1-H) and different from those of Group III (C-X7-C-X23-H-X1-C) (Chen et al., 2017). In addition, Group II is divided into five subgroups, a, b, c, d, and e, according to sequence similarity. Many studies have shown that WRKY transcription factors play important roles in response to biotic stress and regulation of plant growth and development. However, there have been relatively few studies on their other potential roles, especially biotic stress response (Niu et al., 2012).

More recently, in-depth studies have shown that the WRKY family plays an important role in abiotic stress tolerance in plants (Zhou et al., 2015). Fifty-four *OsWRKY* genes respond to salt, cold and drought stress in rice (Ramamoorthy et al., 2008). The expressions of 15 *TaWRKY* genes are reduced by cold, high temperature, NaCl and PEG treatments in *Triticum aestivum* (Wu et al., 2008). There are 18 *AtWRKY* genes responded to salt stress and *AtWRKY25* or *AtWRKY33* overexpression increases salt resistance in *A. thaliana* (Jiang & Deyholos, 2009). *AtWRKY25* also responds to high-temperature stress based on *AtWRKY25* mutant and overexpression transgenic lines (Li et al., 2009). There have been reports that *AtWRKY25*, *AtWRKY26* and *AtWRKY33* oppositely regulate the cooperation between heat shock proteins and the ethylene-activated signaling pathway in *A. thaliana*, and this signaling pathway plays a role in the plant heat stress response (Li et al., 2011). The transcription levels of *AtWRKY6* and *AtWRKY75* are significantly improved under low Pi stress in *Arabidopsis* (Devaiah, Karthikeyan & Raghothama, 2007; Chen et al., 2009). When *HaWRKY76* transgenic lines and wild type *Helianthus annuus* were subjected to water stress, the transgenic plants showed stronger resistance and increased yield (Raineri, Ribichich & Chan, 2015). The expression of 11 *WRKY* genes is upregulated in the roots of *Actinidia chinensis* under water stress, and researchers have speculated that *WRKY* genes may regulate the transcriptional response to water stress in *A. chinensis* (Zhang et al., 2015). *AtWRKY57* overexpression in *Oryza sativa* not only improves drought resistance but also tolerance to salt and PEG in rice (Jiang et al., 2016).

Furthermore, a lot of researchers have suggested that the WRKY family plays a key role in the abscisic acid (ABA) response signal network (Qiao, Li & Zhang, 2016). *LtWRKY21* from the desert plant *Larrea tridentata* activates the promoter of the ABA-induced gene, HVA22, and combines VP1 and ABI5 to form a complex acting downstream of ABI1 to control ABA-related gene expression (Chen et al., 2010). The *AtWRKY1* knockout mutant displays high sensitivity to ABA and low drought resistance (Qiao, Li & Zhang, 2016). ABO3 induces *A. thaliana* to respond to ABA and drought stress (Ren et al., 2010). *AtWRKY40*, *AtWRKY18* and *AtWRKY60* interact with the ABA receptor CHLH/ABAR, to suppress the inhibition of ABA response genes. Among them, *AtWRKY40* is a negative regulator and suppresses the expression of the *ABI5* gene (Shang et al., 2010). *OsWRKY24* and *OsWRKY45* are involved in ABA signal transduction as inhibitory factors, but *OsWRKY72* and *OsWRKY77* regulate positively ABA inducible promoters in rice (Xie, Ruas & Shen, 2005). *CmWRKY10* in transgenic *Chrysanthemum morifolium* improved tolerance to drought stress compared to wild-type plants and the drought tolerance mechanism was associated with ABA pathway (Jaffar et al., 2016). In *Camellia sinensis*, *CsWRKY2* gene has a higher expression level in leaves. The content of ABA increases when *Camellia sinensis* is subjected to drought and cold stresses. *CsWRKY2* gene plays an important role in plant response to cold and drought stress by regulating the downstream ABA signaling pathway (Wang et al., 2016).

During past years, there are plenty *WRKY* genes reported in plants including the dicotyledons *A. thaliana* (74 genes), *Glycine max* (197 genes), *Chaenomeles sinensis* (66 genes), *Citrullus lanatus* (63 genes) and as well as the monocotyledons *O. sativa* (112) , *Sorghum bicolor* (68 genes) , *Ananas comosus* (54 genes) and *Zea mays* (119) (Rushton et al., 2010; Yang et al., 2018; Tao et al., 2018; Ross, Liu & Shen, 2010; Wei et al., 2012). WRKY superfamily has been one of the biggest families in plants (Zhang & Wang, 2005; Rushton et al., 2010). However, the WRKY family genes of Orchidaceae remain lacking and there are still few reports about the field.

Orchidaceae is one of the largest angiosperm families, representing approximately 10% of all angiosperms, and it includes 700 genera and more than 25,000 species worldwide (Dressler, 1993). The researches mainly focus on the flower development and pigment, mycorrhizal symbiosis, crossbreeding and conservation of Orchidaceae plants. With the development of molecular biology technology, MADS-box and MYB family identification and analysis of their functions have had great achievement. Identification of gene family. WRKY superfamily, one of the biggest families in plants, remains in mystery for Orchidaceae plants. *DoWRKY1* was identified from *Dendrobium nobile* in 2015 and activated the downstream reporter genes, *His3* and *Ade2* by yeast one-hybrid analysis. Besides, *DoWRKY1* gene can improve the synthesis of MeJA and chitosan (Zhao et al., 2015). The expression of *DoWRKY5* of *D. nobile* is upregulated by cold stress treatment and *DoWRKY5* can be induced by ABA and source stresses (Jiang et al., 2016). Fifty-two *DoWRKY* genes were identified by fungi and *Dendrobium nobile* transcriptomics and genomic analysis. The *cis*-acting element predictions of DoWRKY promoters show that a promoter includes at least two elements related to stress or hormone response (Wang et al., 2018). When *Oncidium* was grown under high temperature, the leaves were yellowish and spraying different concentrations of salicylic acid (SA) had a protective effect. *OnWRKY1* was cloned from *Oncidium* and it may be associated with heat resistance (Kong, 2017). *Cymbidium goeringii* is a ground-living orchid with small flowers in the family Orchidaceae and genus *Cymbidium*. *C. goeringii* has high ornamental and economic value because of the elegant color and fragrance of its flower and its beautiful leaves. However, *C. goeringii* is vulnerable to abiotic stresses, which lead to a decline in ornamental quality and even plant death, during its growth and development. To address the adverse effects of complex abiotic stress on plant growth and development, plants must regulate their gene expression to adapt to abiotic stress conditions. Transcription factors play an important role in this process (Wei et al., 2016). There are few reports on the field of *C. goeringii*. We identified WRKY family genes from RNA isoform sequencing data of *C. goeringii* and analyzed several WRKY genes. A *CgWRKY* gene had higher expression level among them. In this study, the WRKY gene was identified and cloned from *C. goeringii* and we further explored the response mechanisms of *CgWRKY* genes to abiotic stress, to lay a foundation for further study of the resistance mechanism and to provide a theoretical basis for genetic breeding of *C. goeringii*.

## Materials and Methods

### Plant materials and treatments

The plant material used in the study was *C. goeringii* cv. Song Mei. ‘Song Mei’ is a well-known and recognized traditional variety, and it has a long history of cultivation in China. The flowers of ‘Song Mei’ are typically plum blossom-shaped, and the outer three petals are round similar to the petals of plum trees. The color is green, the petal has a silkworm-moth shape, and the lip has irregular red dots. The fragrance is mild, and the scape is long with one flower. The leaves are dark green, glossy and beautiful (Sun et al., 2012).

‘Song Mei’ was planted in the orchid germplasm resource conservation nursery of Zhejiang Academy of Agricultural Sciences (30°18′N and 120°11′E) under conditions of 70% relative humidity and 22 °C under natural light. The soil was a mixture of bark block, perlite and peat soil. The growing containers of the orchids are 30 cm high approximately to allow better straight root growth. Several two-year-old plants with strong growth were selected for the following treatments. There were three orchid seedlings per pot. The two year old plants have three or four shoots and pseudobulb per pot approximately.

For the high-temperature stress treatment, plants were cultured in an artificial climate chamber at 42 °C, and the leaves were sampled at 0 h, 2 h, 6 h, 12 h, 24 h and 48 h. For the low-temperature stress treatment, plants were cultured in an artificial climate chamber at 4 °C, and the leaves were sampled at 0 h, 2 h, 6 h, 12 h, 24 h and 48 h. For the water stress treatment, roots were completely immersed in water, and the leaves were sampled at 0 h, 2 h, 6 h, 12 h, 24 h and 48 h. For the ABA stress treatment, leaves were sprayed with 100 µM ABA until dripping, and the leaves were sampled at 0 h, 2 h, 6 h, 12 h, 24 h and 48 h (Ren et al., 2010).

The roots, pseudobulbs and leaves were sampled on April 20th, 2018, with the light intensity of 30 µmol/m^2^/s approximately. The floral organs including sepals, petals, lips and column, were sampled from two-year-old ‘Song Mei’ plants on October 10th, 2018, with the light intensity of 50 µmol/m^2^/s approximately.

Each treatment had three groups, and each group included four plants. All samples were cut into approximately 2-cm segments, immediately put into liquid nitrogen and stored at −80 °C.

The transgenic lines used in this study were in the *A. thaliana* ecotype Col-0 background. The plant materials were cultured in an artificial climate chamber under conditions of 80% relative humidity, 22 °C, light intensity of 150 µmol/m^2^/s approximately and a 14 h light/10 h dark photoperiod. The T3 transgenic overexpression plants were used for ABA stress treatment. The leaves of *A. thaliana* were sprayed with 100 µM ABA until dripping and sampled at 0 h, 1 h, 3 h, 6 h and 12 h. All samples were cut into approximately 2-cm segments, immediately put into liquid nitrogen and stored at −80 °C.

### WRKY gene cloning

Total RNA was extracted (MiniBEST Plant RNA Extraction kit, Takara, Dalian) from *C. goeringii*, and first-strand cDNA was synthesized by reverse transcriptase in a volume of 20 µL (PrimeScript RT Master Mix, Takara, Dalian). cDNA was diluted to a suitable concentration for PCR. The primers were designed by Oligo6 software according to the sequence of the *CgWRKY* gene (Table 1), and the full-length *CgWRKY* gene was cloned by RT-PCR. The PCR procedure was as follows: 94 °C for 3 min; 32 cycles of 98 °C for 10 s, 56 °C for 15 s, 72 °C for 30 s; and 72 °C for 5 min. The PCR products were detected by 1.5% agarose gel electrophoresis. The amplified fragment was purified and connected to the pEASY-Blunt vector. The vector was then transformed into Trans5 *α* cells, and the positive clones were tested.

**Table 1:** Primers used for cloning, RT-qPCR and double enzyme digestion assays.

Primer name	Primer sequence (5′ → 3′)
CgWRKY57-cloning-F	ATGGGATCTCAGGAGGAGG
CgWRKY57-cloning-R	CTAACCTTGAAACAGATCGGT
CgWRKY57- RT-qPCR-F	CTGCTCTACTCCAAATCGG
CgWRKY57- RT- qPCR-R	GGATCAGCCACTCGATTAA
CgWRKY57-SmaI-F	CCCGGGATGGGATCTCAGGAGGAGG
CgWRKY57-SnaBI-R	TACGTAACCTTGAAACAGATCGGTATG
35S-F	GACGCACAATCCCACTATCC

### Sequence analysis

Online prediction of the physical and chemical properties of the amino acid sequence, including molecular weight and isoelectric point, was conducted by the ExPASy server (http://web.expasy.org/compute_pi/) (Wei et al., 2016). Online prediction of protein subcellular localization was conducted by WOLF PSORT (http://www.genscript.com/psort/wolf_psort.html) (Horton et al., 2007). Online prediction of conserved domains was conducted by SMART (http://smart.embl-heidelberg.de/) (Lvica & Peer, 2018). The multiple sequence alignment was analyzed by Clustal W (http://www.clustal.org/clustal2/) and DNAMAN5.0 software (Lynnon Biosoft, https://www.lynnon.com/dnaman.html) (Larkin et al., 2007). The amino acid sequences of AtWRKY and OsWRKY proteins were downloaded from TAIR 9.0 (http://www.arabidopsis.org/index.jsp) and the Plant Transcription Factor Database (http://planttfdb.cbi.pku.edu.cn/). The phylogenetic tree was constructed by MEGA 7.0 software with the neighbor-joining method (NJ) and a bootstrap value of 1000 (Kumar, Stecher & Tamura, 2016).

### Real-time quantitative PCR analysis

RT-qPCR was performed using SYBR Green (Takara) on an ABI qPCR system ABI (StepOne Plus, USA) in a volume of 10 µL. The reaction system included 5 µL of 2 × SYBR Green Premix Ex Taq (Tli RNaseH Plus), 0.2 µL of 50 × ROX Reference Dye, 0.2 µL of forward and reverse primers (10 µM), 1 µL of cDNA template and 3.4 µL of RNase-Free ddH_2_O. The procedure was as follows: 95 °C for 3 min; and 40 cycles of 95 °C for 3 s and 60 °C for 30 s. The *18S RNA* of *C. goeringii* was used as the reference gene, and the data were analyzed by the 2^−ΔΔ*Ct*^ method. All experiments included three independent biological replicates and three technical replicates. The primer sequences used for RT-qPCR are listed in Table 1.

### PBI121-CgWRKY57 vector construction and plant transformation

The coding sequence (CDS) of *CgWRKY57* was cloned into the PBI121 vector by Sma I/SnaB I double digestion to generate the 35S::CgWRKY57 vector. The primer sequences used for double digestion are listed in Table 1. The recombinant plasmid was introduced into *Agrobacterium tumefaciens* strain EHA105 and transformed into *A. thaliana* by the floral dip method. The inflorescences were infected for 60 s and incubated in the dark for 24 h. The resulting plants were screened for resistance until the T3 transgenic plants were obtained.

### ABA stress treatment for T3 transgenic plants

Forty-day-old wild-type (WT) Col-0 plants and T3 transgenic plants were cultured in an artificial climate chamber under conditions of 22 °C and 150 µmol/m^2^/s. The plants were sprayed with 100 µM ABA and incubated for 24 h. The leaves were sampled at 0 h, 1 h, 3 h, 6 h and 12 h, and they were stored at −80 °C.

### Determination of germination rate and root length of transgenic plants

Fifty WT Col-0 and T3 transgenic seeds were disinfected and cultured in vitro on culture media with 0 µM ABA, 1 µM ABA and 2 µM ABA, respectively. Three repeated experiments were conducted. After vernalization for 2 days at 4 °C, culture media were placed in an artificial climate chamber under conditions of 80% relative humidity, 22 °C, light intensity of approximately 150 µmol/m^2^/s, and a 14 h light/10 h dark photoperiod. The in vitro seed germination rate was recorded on the 6th day, and the root length was measured on the 12th day in the 0 µM ABA treatment. The criterion for germination was seeds showing 2 mm long white roots.

## Results

### *CgWRKY57* cloning and analysis of protein physical and chemical properties

The *CgWRKY57* sequence was selected from *C. goeringii* transcriptome data. *CgWRKY57* gene was annotated in the Gene Ontology (GO) (Cellular component: nucleus, GO:0005634; Molecular function: transcription factor activity, GO:0003700; sequence-specific DNA binding, GO:0043565), Pfam (PF03106.15) and Clusters of Orthologous Groups (COG) (Functional categories: K: Transcription) databases. Primers were designed to amplify the *CgWRKY57* sequence, and the cDNA of *C. goeringii* was used as a template for PCR. The PCR product was detected by 1% agarose gel electrophoresis, and the amplified fragment was found to match the sequenced fragment (Fig. S1A). The *CgWRKY57* ORF is 912 bp in length. The CgWRKY57 protein is acidic, and its molecular weight and isoelectric point were predicted by the ExPASy server. When predicting protein hydrophobicity, if the total average hydrophilicity is positive, the protein is hydrophobic, and larger positive values indicate stronger hydrophobicity. If the total average hydrophobicity is negative, the protein is hydrophilic, and larger negative values indicate stronger hydrophilicity (Dai et al., 2014). Therefore, CgWRKY57 is a hydrophilic protein. Guruprasad assigned a weight value of instability to each of the 400 different dipeptides (DIWV) to compute an instability index and proposed that a protein whose instability index is smaller than 40 is predicted as stable, a value above 40 predicts that the protein may be unstable (Guruprasad, Reddy & Pandit, 1990). CgWRKY57 was predicted to be an unstable protein because its instability index was greater than 40. The CgWRKY57 protein has no signal peptide according to the results of SignalP 4.1 (Fig. S1B). The CgWRKY57 protein is localized outside the membrane without a transmembrane region according to transmembrane helix prediction (Fig. S1C). The CgWRKY57 protein is located to the nucleus and may function intracellularly according to the results of the subcellular localization prediction by PSORT online software (Table 2). The secondary structure of CgWRKY57 was analyzed by SOPMA software and consisted of 62.71% random coil, 19.80% *α*-helix, 13.86% extended strand and 3.63% *β*-fold structure (Fig. S1D).

**Table 2:** Prediction of CgWRKY57 protein subcellular localizations.

Subcellular structures	CgWRKY57
Nuclear	11
Mitochondrial matrix	2
Chloroplast	1

**Figure 1:**
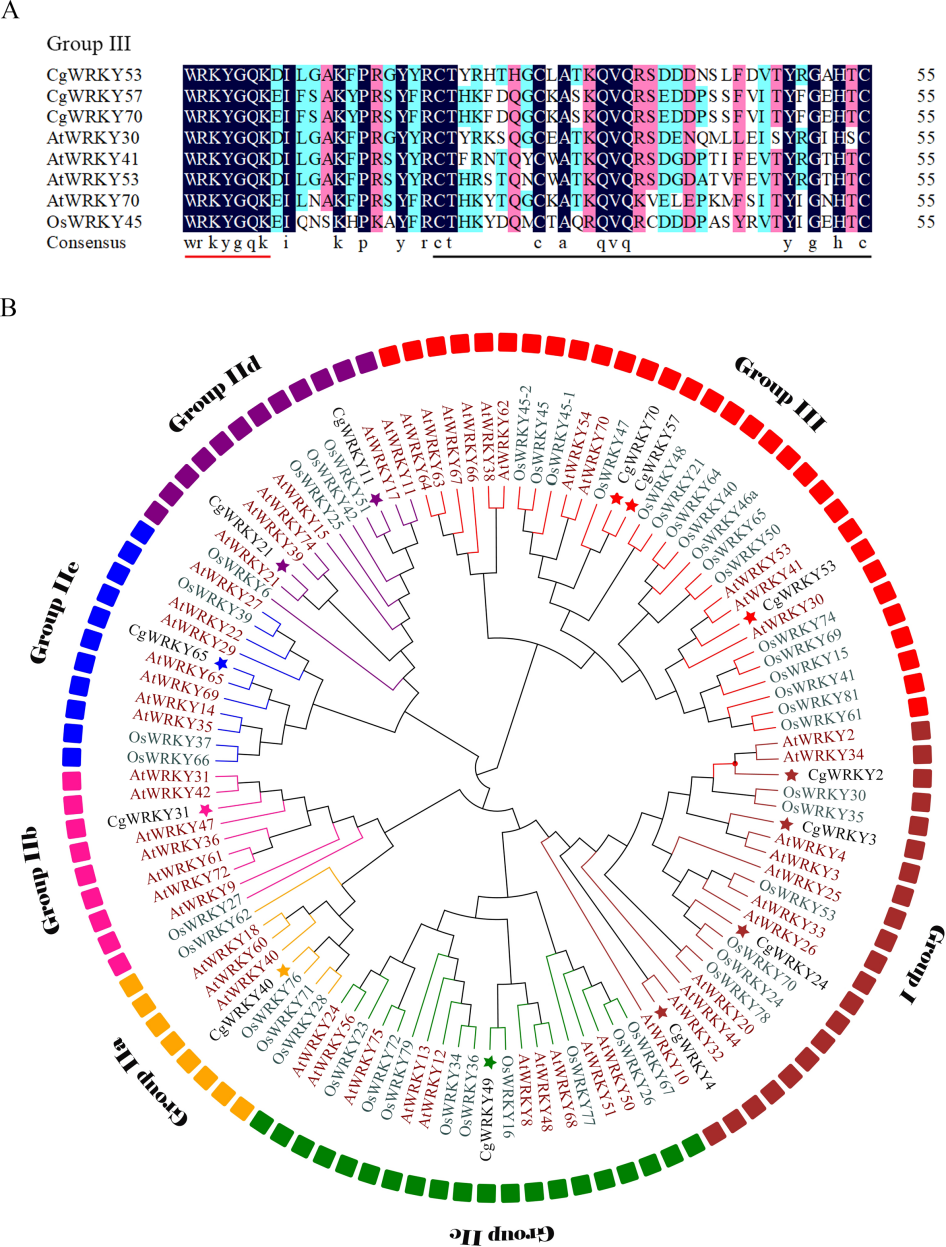
Sequence alignment and phylogenetic tree analysis of WRKY domains from *C. goeringii*, *O. sativa*, and *A. thaliana*. (A) Amino acid sequence alignment of conserved WRKY domains in *C. goeringii*, *O. sativa*, and *A. thaliana*; (B) Phylogenetic tree analysis of CgWRKY57; Black font, *C. goeringii*; Brown font, *A. thaliana*; Green font, *O. sativa*. The phylogenetic tree was constructed by MEGA 7.0 software with the neighbor-joining (NJ) method and a bootstrap value of 1000. The phylogenetic tree was divided into seven subgroups named Groups I, IIA–E, and III.

### Sequence and phylogenetic tree analysis

CgWRKY57 has the typical “WRKYGQK” domain and zinc finger domain of the WRKY family according to the SMART online software analysis. A sequence alignment showed that CgWRKY57 has a WRKY domain and a zinc finger domain (C2HC) and that it belongs to Group III (Fig. 1A). To study the phylogenetic relationships among the WRKY proteins of *C. goeringii*, *O. sativa*, and *A. thaliana*, a phylogenetic tree was constructed (Fig. 1B). CgWRKY57 has a close relationship with OsWRKY47, and we hypothesized that they have similar biological functions. Group I genes are reportedly the most ancient WRKY family members, and they may have retained or lost the WRKY domain in the N-terminus. Group II and Group III are the offspring of Group I, and they only have a WRKY domain in the N-terminal (Zhang & Wang, 2005).

Phylogenetic tree analysis not only shows the phylogenetic relationships among family members but also provides potential support for functional similarity. WRKY proteins with the same function will group together. For example, *AtWRKY54* and *AtWRKY70* of Group III are in the same clade, and both regulate leaf senescence (Besseau, Li & Palva, 2012). Similarly, *AtWRKY18*, *AtWRKY40* and *AtWRKY60* of Group IIa are in the same branch and negatively regulate the transcription of receptor-like kinase CRK5 (Lu et al., 2016).

### Expression patterns of *CgWRKY57* in different tissues and under abiotic stresses

**Figure 2:**
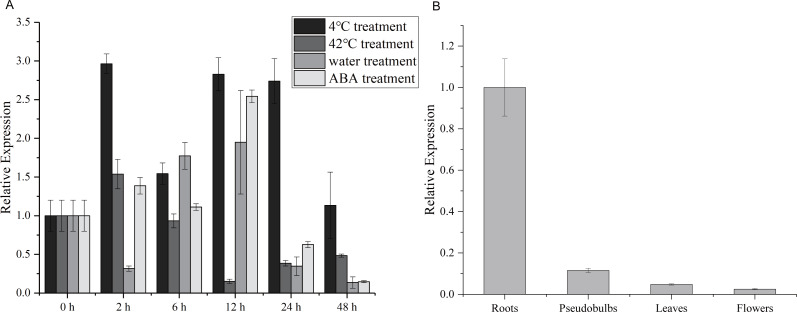
Expression patterns of *CgWRKY57* in different tissues and under abiotic stresses of *C. goeringii*. (A) Expression patterns of *CgWRKY57* in 4 °C, 42 °C, waterlogging and ABA treatment; (B) Expression patterns of *CgWRKY57* in different tissues.

To explore the potential role of the *CgWRKY57* gene in response to stress and in four different tissues, RT-qPCR was used to analyze the dynamics of *CgWRKY57* under low-temperature, high-temperature, water and ABA stress as well as the expression of *CgWRKY57* in four tissues (roots, pseudobulbs, leaves and flowers). The leaves of *C. goeringii* appeared yellow and aged after 48 h of high temperature or water immersion, and the expression analysis showed that the expression of *CgWRKY57* was induced by high-temperature and water stresses (Fig. S2). The results showed that *CgWRKY57* was expressed in all the organs and showed different expression levels in different organs (Fig. 2). The expression of *CgWRKY57* was highest in roots (*P* < 0.05), suggesting that *CgWRKY57* may be related to root development. The expression of *CgWRKY* 57 was relatively high at 2 h, 12 h and 24 h after low-temperature treatment; it decreased at 48 h but was still higher than that at 0 h. The expression of *CgWRKY57* was enhanced at 2 h after high-temperature treatment but then decreased continuously. The expression of *CgWRKY* 57 was upregulated during water treatment at 6 h and 12 h and was highest at 12 h, while its expression at 48 h was similar to that at 0 h. After ABA treatment, the expression of *CgWRKY* 57 showed a downward trend with its highest point at 12 h and its lowest at 48 h. Remarkably, the expression level of *CgWRKY57* changed most obviously after ABA stress, and it had a downward trend. Thus, it is predicted that *CgWRKY57* responds most strongly to ABA stress.

**Figure 3:**
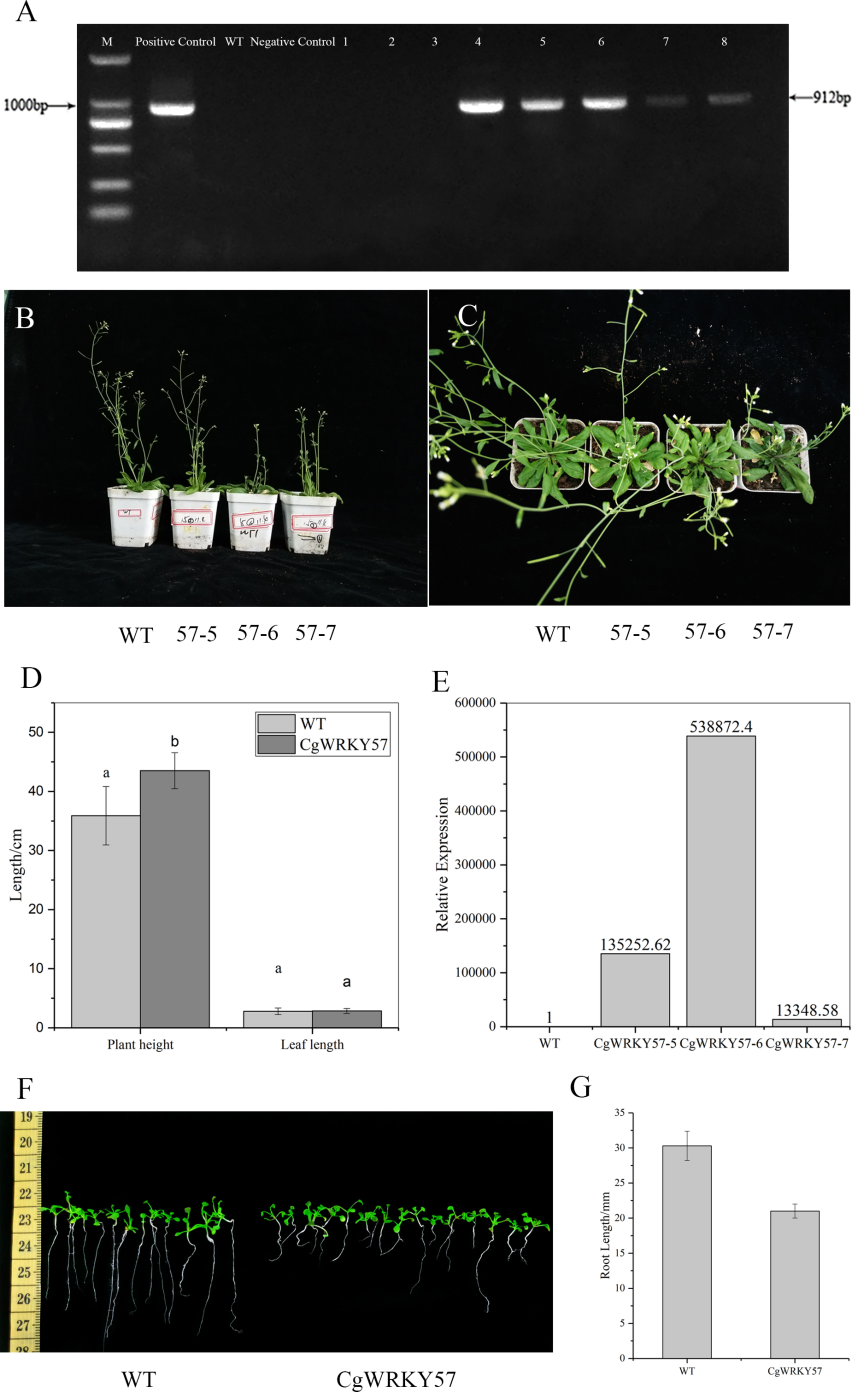
PCR identification and phenotype observation of T3 overexpression *CgWRKY57* transgenic plants. (A) The PCR identification of overexpression *CgWRKY57* transgenic plants; (B) side view of overexpression *CgWRKY57* transgenic plants and WT plants; (C) top view of overexpression *CgWRKY57* transgenic plants and WT plants; (D) the plant height and leaf length of overexpression *CgWRKY57* transgenic plants and WT plants; (E) the expression level of overexpression *CgWRKY57* transgenic plants and WT plants; (F, G) root length measurements of transgenic plants overexpressing *CgWRKY57*.

### Identification and phenotype observation of transgenic plants overexpressing *CgWRKY57*

PCR detection was conducted with the 35S forward primer and *CgWRKY57* reverse primer using the DNA from T3 transgenic and WT *Arabidopsis* as well as the PBI121-CgWRKY57 plasmid and water as templates. The results showed that the target product was not amplified from WT *Arabidopsis* DNA, while T3 transgenic plant DNA allowed amplification. These results indicated that *CgWRKY57* was successfully transformed into *Arabidopsis* (Fig. 3A).

Seven-day-old *Arabidopsis* seedlings were transplanted into pots and cultured in a light incubator. The phenotype of the transgenic plants was observed after 30 days (Figs. 3B and 3C). Compared to WT *Arabidopsis*, transgenic plants overexpressing *CgWRKY57* grew slowly and were dwarfed. The leaf shape showed no change, but leaf etiolation was obvious (Fig. 3D). The plant height of WT *Arabidopsis* was about 35.90 cm, but the plant height of overexpressing *CgWRKY57* was 43.50 cm (Fig. 3D). The of transgenic plants flowered in 26 days after being transplanted into pots and the inflorescence emerged approximately five days later than that of WT *Arabidopsis*. Then, we detected the expression level of overexpressing *CgWRKY57* and WT *Arabidopsis* plants (Fig. 3E). The results indicated the *CgWRKY57* expression was significantly enhanced in transgenic plants than WT plants.

To measure the root length of transgenic plants overexpressing CgWRKY57, transgenic and WT *Arabidopsis* were in vitro germinated on MS solid culture medium, and their root lengths were recorded 10 days later. The results showed that the root length of the WT plants was 31 mm and that of the transgenic plants was 20 mm. The roots of the WT *Arabidopsis* seedlings were long and straight, and those of the transgenic plants were short and curved. The root length of WT *Arabidopsis* was significantly longer than that of the transgenic plants (Figs. 3F and 3G).

### Expression analysis and germination rate of transgenic plants overexpressing *CgWRKY57* under ABA stress

**Figure 4:**
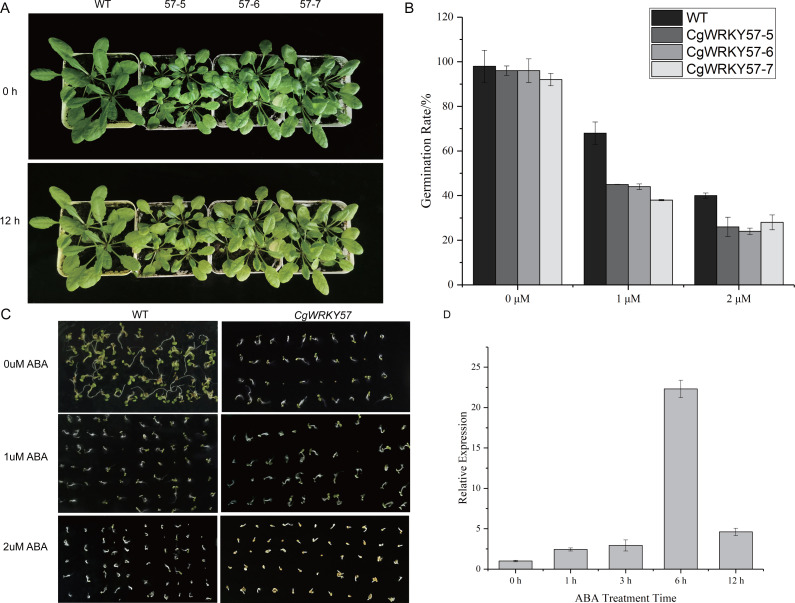
Phenotype observation and RT-qPCR analysis of transgenic plants overexpressing *CgWRKY57* under ABA stress treatment. (A) Top view of overexpression *CgWRKY57* transgenic plants and WT plants under ABA stress treatment; (B) the expression level of overexpression *CgWRKY57* transgenic plants under ABA stress treatment; (C, D) germination rates of transgenic plants overexpressing *CgWRKY57* under ABA treatment.

To further study the function of *CgWRKY57* under ABA stress, *CgWRKY57* transgenic and WT *Arabidopsis* plants were treated with ABA. At 0 h of ABA treatment, the leaves of the transgenic *Arabidopsis* plants were yellow, smaller and more wilted than those of the WT plants. At 12 h, the yellow leaves of the transgenic *Arabidopsis* plants were yellower and weaker than those of the WT plants (Fig. 4A). The expression of *CgWRKY57* was detected by RT-qPCR. The results showed that *CgWRKY57* was not expressed in WT plants and that its expression in transgenic *Arabidopsis* was high. The expression level was the lowest at 0 h and increased significantly at 6 h, indicating that *CgWRKY57* was responsive to ABA (Fig. 4B).

Then, we investigated the germination rate of transgenic plants overexpressing *CgWRKY57* under ABA stress. *CgWRKY57*-overexpressing transgenic and WT *Arabidopsis* were in vitro germinated on culture media with 0 µM ABA, 1 µM ABA and 2 µM ABA. After 7 days, the germination rate was determined. The germination criterion was that the white root length reached 1/2 of the seed length. The germination rates of the transgenic and WT *Arabidopsis* seeds were both more than 90%. The germination rate of the WT seeds was slightly higher than that of the *CgWRKY57* transgenic seeds, but the difference was not significant. Under the 1 µM ABA stress treatment, however, the germination rates of both the WT and transgenic *Arabidopsis* seeds decreased significantly. The germination rate of WT *Arabidopsis* seeds was more than 65% and significantly higher than that of the *CgWRKY57* transgenic seeds, which was approximately 40%. Under the 2 µM ABA stress treatment, the germination rate of WT *Arabidopsis* seeds decreased to 40% and that of *CgWRKY57* transgenic seeds decreased to approximately 25%. Overall, the germination rate of *CgWRKY57* transgenic *Arabidopsis* seeds decreased more than that of WT seeds compared to the germination rates in the 0 µM ABA treatment, indicating that the germination of *CgWRKY57*-overexpressing seeds was affected more by ABA (Figs. 4C and 4D).

## Discussion

*C. goeringii* has high ornamental value, but it is vulnerable to abiotic stresses and its ornamental quality will be affected. WRKY transcription factors play important roles in plant growth, development and stress response and have been identified from a variety of plants (Yang et al., 2018). However, there are few reports on the field of *C. goeringii*. In this study, a CgWRKY protein was obtained and we analyzed the structural characteristics by comparing its conserved WRKY domain to those of previously characterized proteins and constructing a phylogenetic tree. The results indicated that the conserved WRKY domain is indispensable for *CgWRKY* genes. Many studies have also indicated that the highly conserved WRKY domain is the most prominent feature of the WRKY family (Huang et al., 2012). Many species have variants of the WRKYGQK sequence, such as WRKYGKK, WSKYEQK and WRKYSEK (Mohanta, Park & Bae, 2016). However, the core sequence of the CgWRKY domain contains no mutations, suggesting that the CgWRKY domain is highly conserved and that its amino acid sequence did not undergo a large amount of change during evolution. In addition, the phylogenetic analysis showed that *CgWRKY57* belongs to Group III of the WRKY family. The zinc finger domains of different groups of WRKY proteins vary greatly in sequence length and structure, and the zinc finger domain of Group III is C2HC, indicating that the different groups in the WRKY family have undergone different selection pressures and evolution patterns (Bi et al., 2016; Wu, 2005). Phylogenetic analysis not only shows the phylogenetic relationships among members but also provides potential support for functional similarity (Jiang et al., 2014). *CgWRKY57* and *OsWRKY47* cluster together in a clade, and we hypothesized that they have similar biological functions.

Gene expression patterns provide clues for functional research, and the WRKY family, which is highly expressed in plant tissues, is usually involved in regulating plant growth and development (Yu et al., 2012). *TRANSPARENT TESTA GLABRA2* (*TTG2*) encoding a WRKY TF of *Arabidopsis* is strongly expressed in trichomes and seed coat throughout their development (Johnson, Kolevski & Smyth, 2002).12 of the 37 members of WRKY TF family in *Arabidopsis* are found in localized expression domains of roots, indicating a possible specialized role in root cell maturation (Birnbaum et al., 2003). It has been previously reported that *SmWRKY2* and *SmWRKY24* of *Salvia miltiorrhiza* are highly expressed in roots with many root hairs, indicating that this gene is related to root morphogenesis (Li et al., 2015). *OsWRKY74* is highly expressed in the root, and the Pi content of *OsWRKY74*-overexpressing plants is significantly higher than that of control plants under phosphate deficiency indicating that *OsWRKY74* promotes phosphate absorption by the root (Dai, 2016). As a positive regulator of Pi, *AtWRKY75* also affects the development of roots. When *AtWRKY75* gene is inhibited, the number of lateral roots and root hairs increases (Devaiah, Karthikeyan & Raghothama, 2007). In the study, a WRKY gene were cloned from *C. goeringii*, named Cg*WRKY57*. And *CgWRKY57* was highly expressed in roots. Above all, these findings suggest that WRKY family is related to the development of roots.

Plants defend against environmental stresses through a complex regulatory network in vivo, and WRKY transcription factors play important roles in this network (Rushton et al., 2010). Plants often encounter water stress, which affects growth and development and reduces oxygen, due to irrigation throughout their life cycles (Voesenek & Bailey-Serres, 2015). Notably, the expression patterns of *WRKY* genes are similar under different stresses in many studies. The expression of *CsWRKY46* in *Cucumis sativus* is upregulated under cold stress and ABA treatment (Ying et al., 2016), and the expression of *SlWRKY3* in *Solanum lycopersicum* is induced under NaCl, KCl and drought stresses (Hichri et al., 2017). *AtWRKY6* plays roles in pathogen defense and aging, and *AtWRKY70* is involved in regulating plant growth, drought response and cell death (Robatzek & Somssich, 2010). It has been reported that *AtWRKY54* and *AtWRKY70* negatively regulate the response to necrotrophic pathogens in *Arabidopsis*. Increased SA content in the *wrky54wrky70* double mutant leads to the accumulation of H_2_O_2_ and strengthening of the cell wall, which improves resistance to necrotrophic bacteria in *Arabidopsis* (Li, Zhong & Palva, 2017). Another study has found that the *wrky54wrky70* double mutant shows significantly greater tolerance to osmotic stress, indicating that *WRKY70* and *WRKY54* are negative regulators of stomatal closure and regulate osmotic stress tolerance in plants (Li et al., 2013). In this study, we analyzed the expression patterns of *CgWRKY57* under four stress treatments, including high-temperature, low-temperature, water, and ABA stresses. *C. goeringii* has fleshy roots, which rot easily under water stress, and this problem can even lead to plant death. The expression of *CgWRKY57* under water stress was upregulated at 6 h and 12 h. The expression of *AtWRKY22* is also induced rapidly under water stress (Hsu et al., 2013). The AtWRKY22 protein binds to the promoter of TREHALASE1 (TRE1) and inhibits its expression, enhancing water stress resistance by affecting the stomata (Hsu et al., 2013). In addition, *AtWRKY22* regulates the expression of *MYB15*, *PUB24* and *ACS7*, which are related to plant immune responses (Cai et al., 2013). Immune responses in *A. thaliana* can be caused by water stress, and *AtWRKY22* enhances the ability to resist pathogen infection. Both *CgWRKY57* and *AtWRKY22* belong to the WRKY transcription factor family and have the same WRKY domain. *CgWRKY57* belongs to Group III and *AtWRKY22* belongs to Group IIe of WRKY family. Thus, we hypothesized that many WRKY family proteins are likely to have similar functions. The expression of *CgWRKY57* was downregulated under high-temperature but upregulated under low-temperature stress, indicating that *CgWRKY57* was induced by low temperature and inhibited by high temperature. In addition, the degree and timing of *CgWRKY57* expression in response to different stresses were significantly different. the expression of *CgWRKY57* rapidly reached a peak (at 2 h) under low-temperature treatment, but it did not change at 2 h under water stress, instead increasing rapidly at 6 h and reaching a peak at 12 h. The ABA stress hormone plays a key role in response to abiotic stresses, and WRKY proteins are a key node in the ABA response signal network, acting as activators or inhibitors in this signaling pathway (Qiao, Li & Zhang, 2016; Chi et al., 2013). AtWRKY40 inhibits the expression of ABI5 in response to ABA and negatively regulates the ABA signal transduction pathway by interacting with ABAR, which is a negative regulatory factor (Shang et al., 2010). The expression of *CgWRKY57* in *C. goeringii* was inhibited by ABA treatment, consistent with previous research. Thus, we speculated that *CgWRKY57* might also regulate the expression of genes related to ABA stress. These results suggested that *CgWRKY57* responded to four abiotic stresses, indicating that *CgWRKY57* plays an important role in adaptation to environmental stresses in *C. goeringii* and a single gene can regulate responses to various stresses. Moreover, the results lay a foundation for further functional study.

WRKY transcription factors constitute one of the largest regulatory protein families in plants and play an important role in response to abiotic stresses. Song et al. transferred *OsWRKY72* into *Arabidopsis*, obtained *35S-OsWRKY72* transgenic *Arabidopsis* plants and observed their phenotypes. The results showed that the transgenic plants also have abnormal phenotypes, such as leaf senescence and dwarfing, indicating that *OsWRKY72* has a strong impact on *Arabidopsis* morphology (Song et al., 2010). Some studies have shown that *AtWRKY53* is significantly upregulated in the early stage of leaf senescence and that its overexpression in transgenic *Arabidopsis* plants promotes senescence, while silencing of *AtWRKY53* delays leaf senescence (Miao et al., 2004). Besseau et al. knocked out *AtWRKY54* and *AtWRKY70* to obtain an *atwrky54-atwrky70* double mutant and found that the leaves of these plants show premature senescence. This result indicated that there might be a synergistic effect between *AtWRKY70* and *AtWRKY54* in the negative regulation of leaf senescence (Besseau, Li & Palva, 2012). To further study the function of the *CgWRKY57* transcription factor, *CgWRKY57*-overexpressing plants were obtained by the floral dip method. *CgWRKY57*-overexpressing transgenic plants were dwarfed, and their leaves appeared withered and yellow comparing with WT *Arabidopsis*, suggesting that overexpression of this gene might positively regulate the plant senescence process. The root length of *CgWRKY57* transgenic plants was significantly shorter than that of WT plants, indicating that *CgWRKY57* inhibits *Arabidopsis* root growth.

ABA is a stress hormone with an important role in plant responses to abiotic stress. Previous studies have shown that WRKY proteins positively or negatively regulate ABA signal transduction (Mohanta, Park & Bae, 2016). *LtWRKY21* of *Larrea tridentata* positively regulates the ABA signaling pathway (Zou et al., 2004). Transient expression analysis has shown that *OsWRKY24* and *OsWRKY45* inhibit ABA signal transduction but that *OsWRKY72* and *OsWRKY77* positively regulate inducible promoters related to ABA (Xie, Ruas & Shen, 2005). As shown by inserting the *atwrky2* T-DNA into the mutant, *AtWRKY2* is part of a negative feedback loop in seed germination and later development mediated by ABA (Jiang & Yu, 2009). Another study has shown that *AtWRKY18* and *AtWRKY60* increased ABA sensitivity, inhibiting seed germination and root growth. Moreover, overexpression of *AtWRKY18* and *AtWRKY60* increased sensitivity to salt and osmotic stresses (Chen et al., 2010). Transgenic plants overexpressing *CgWRKY57* turned yellow under ABA stress and the expression of *CgWRKY57* in transgenic plants increased significantly at 6 h of ABA stress. And, the germination rates of both WT and transgenic plants decreased significantly with increasing ABA concentration, and the germination rate of the latter was lower than that of the former. Together, the above results showed that *CgWRKY57* has a strong response to ABA and positively regulates the inhibition of ABA. Therefore, it is inferred that *CgWRKY57* is sensitive to ABA and may be involved in ABA signal transduction as an inhibitor, but the response mechanism still requires further study.

## Conclusion

In this study, the *CgWRKY57* gene of *C. goeringii* was comprehensively analyzed. *CgWRKY57* belongs to Group III according to the bioinformatics analysis, and its homology and phylogenetic relationships with rice and *Arabidopsis* WRKY genes provide valuable clues to its function. The expression patterns of *CgWRKY57* in different tissues of *C. goeringii* and under various abiotic stresses were determined, and the function of *CgWRKY57* was characterized in *Arabidopsis*. These results lay a foundation for further study of the function of *CgWRKY57* and provide ideas for future research on breeding *C. goeringii* for adversity resistance.

##  Supplemental Information

10.7717/peerj.10982/supp-1 File S1 CgWRKY57 sequence

10.7717/peerj.10982/supp-2 Supplemental Information 1 Germination tate data

10.7717/peerj.10982/supp-3 Supplemental Information 2 RT-qPCR raw data

10.7717/peerj.10982/supp-4 Supplemental Information 3 Root length data

10.7717/peerj.10982/supp-5 Figure S1 *CgWRKY57* cloning (A) from *C. goeringii* and its physical and chemical properties analysis including signal peptide prediction (B), transmembrane helix prediction (C), and secondary structure prediction (D)

10.7717/peerj.10982/supp-6 Figure S2 Phenotype observation of *C. goeringii* under 42 °C treatment (A) and water treatment (B) after 48 h

## Additional Information and Declarations

### DNA Deposition

The following information was supplied regarding the deposition of DNA sequences: The sequences described here are available in the Supplemental Files and at GenBank: MW228372.

### Data Availability

The following information was supplied regarding data availability: Raw data are available in the Supplemental Files.
