# Supplementary figures and images for: Functional analysis of CgWRKY57 from Cymbidium goeringii in ABA response
Source: PeerJ. 2021 Feb 23;9:e10982. doi: 10.7717/peerj.10982 (PMC7908890; doi:10.7717/peerj.10982)

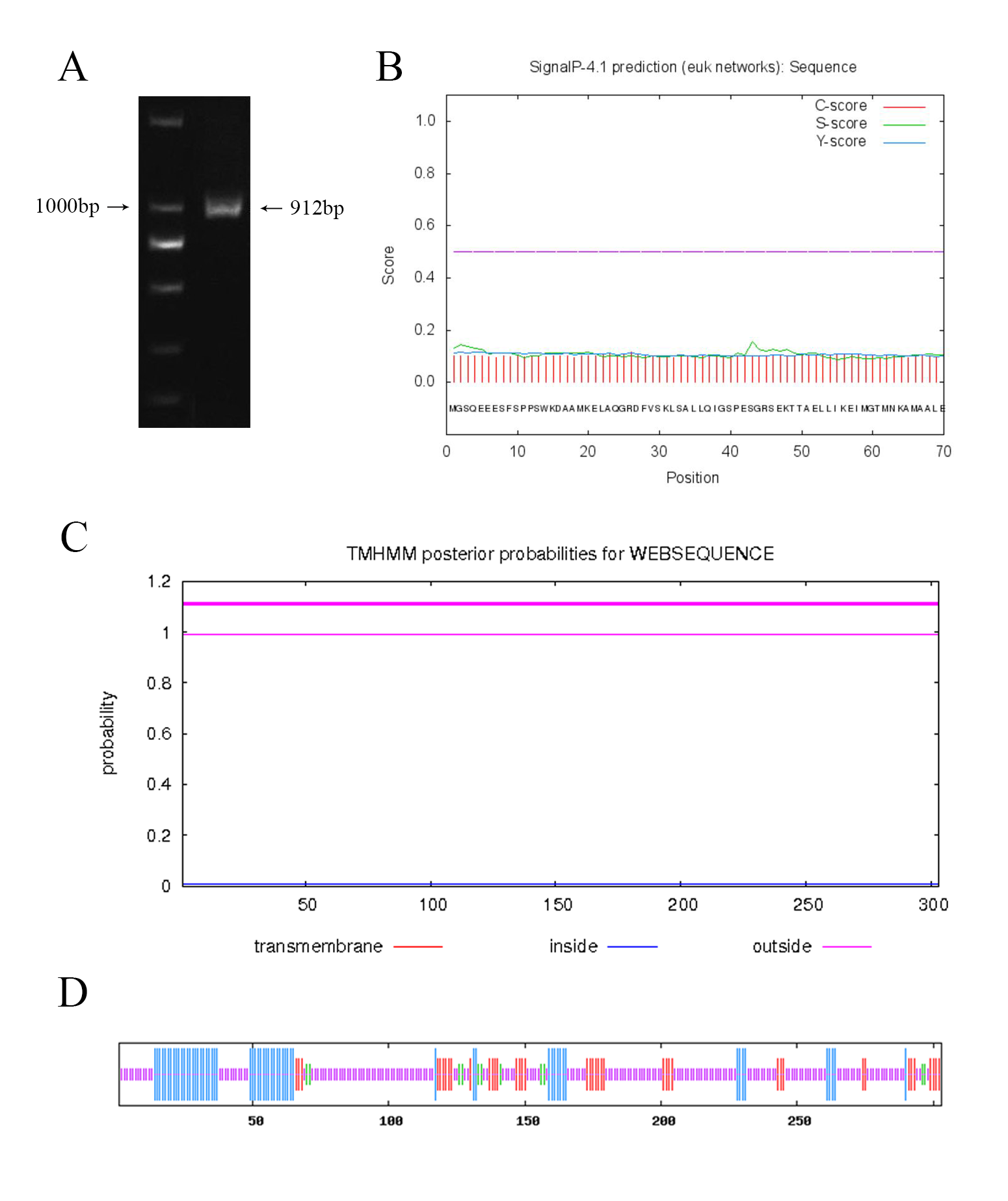

Supplement: Figure S1 [file peerj-09-10982-s005.png]

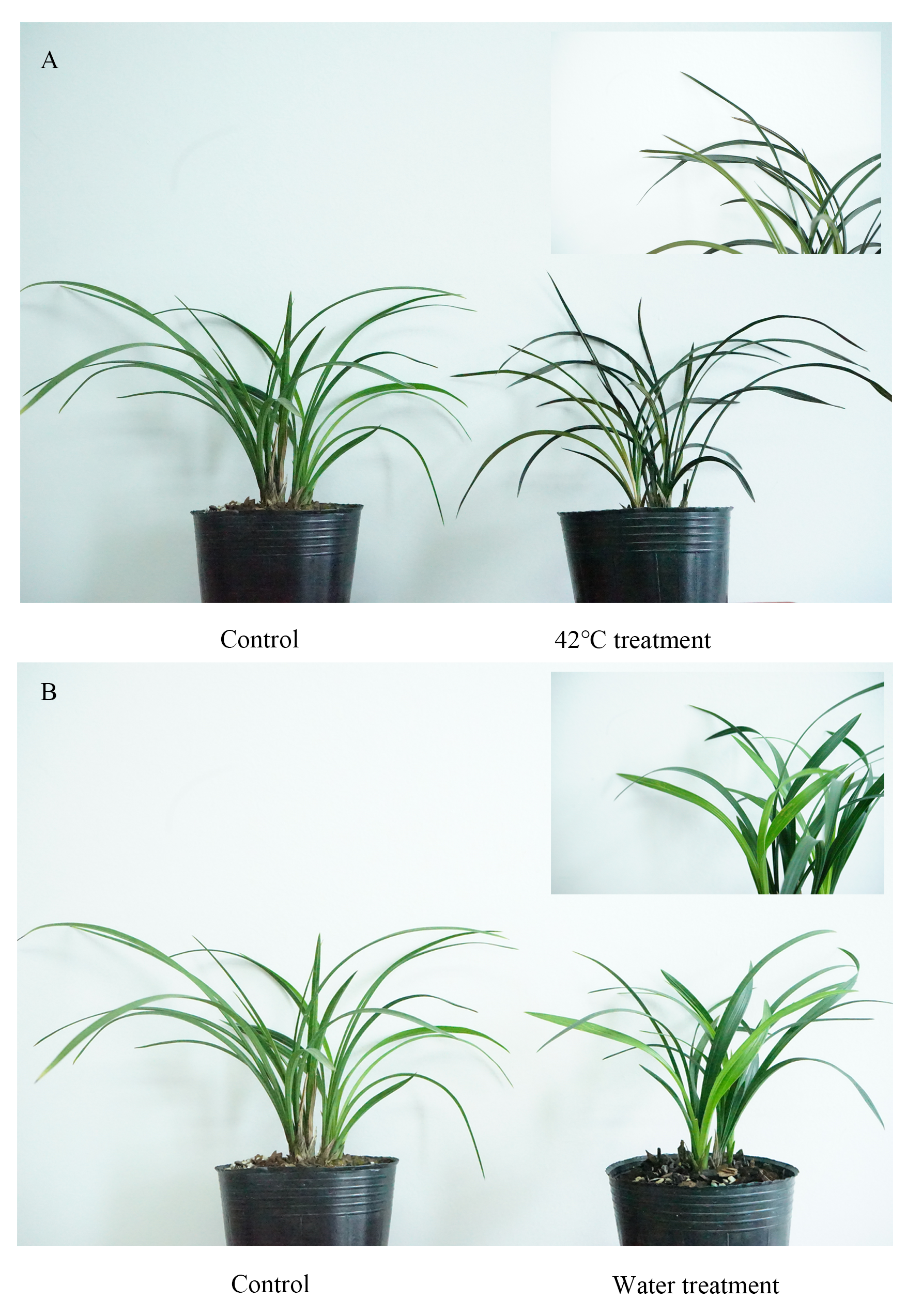

Supplement: Figure S2 [file peerj-09-10982-s006.png]
